# Microbiome analysis reveals dynamic changes of gut microbiota in Guizhou horse and Dutch Warmblood horses

**DOI:** 10.3389/fmicb.2025.1562482

**Published:** 2025-03-12

**Authors:** Yanfang Lan, Yaonan Li, Yang Wang

**Affiliations:** School of Physical Education and National Equestrian Academy, Wuhan Business University, Wuhan, China

**Keywords:** gut microbiota, horse, bacteria, fungi, PCoA, abundance

## Abstract

In recent years, the importance of gut microbiota in digestive absorption, metabolism, and immunity has garnered increasing attention. China possess abundant horse breed resources, particularly Guizhou horses, which play vital roles in local agriculture, tourism, and transportation. Despite this, there is a lack of comparative studies on the gut microbiota of native Guizhou horses (GZH) and imported Dutch Warmblood horses (WH). To address this gap, fecal samples were collected from both GZH and WH, and 16S rRNA high-throughput sequencing was utilized to analyze the differences in their gut microbiota. The results indicated that compared with GZH, the abundance of the gut bacterial community in WH was significantly higher, whereas the abundance of the gut fungal community was lower. Furthermore, PCoA-based scatter plot analysis demonstrated distinct differences in the structure of gut bacteria and fungi between the two breeds. While both types of horses share similar major bacterial and fungal phyla, significant differences were observed in numerous bacterial and fungal genera. Moreover, functional predictions of gut bacterial communities suggested that WH exhibit a more robust digestive system and enhanced glycan biosynthesis and metabolism capabilities. This is the first report on the comparative analysis of the gut microbiota in GZH and WH. The results emphasize the significant differences in gut microbiota among various horse breeds and offer valuable insights into the composition and structure of gut microbiota in different horse breeds.

## Introduction

Gut microbiota has gained significant attention in recent years due to its vital roles in host health and physiological functions (Guo et al., 2021; Li S. et al., 2023). Research has shown that gut microbiota is closely associated with nutrient absorption, immune system development, metabolism, and the maintenance of the intestinal mucosal barrier (Bao et al., 2022; Cheng et al., 2019). Moreover, recent studies involving gut microbiota have also revealed its key roles in intestinal epithelial differentiation, skeletal development, and colonization resistance (Beaumont et al., 2022; Zhang Y. et al., 2022). However, the composition and diversity of gut microbiota are influenced by intrinsic characteristics and external factors. Intrinsic characteristics such as gender, age, and species are considered to be the main factors affecting the gut microbial composition and structure (Santos-Marcos et al., 2018). Additionally, external factors, including diet, environmental factors (heavy metals, microplastics, and pesticides), and geographical environment, are the primary driving forces cause gut microbial dysbiosis (Cheng et al., 2021; Meng et al., 2020). Gut microbial dysbiosis is mainly characterized by significant changes in the microbial composition and structure, and it has been demonstrated to be a core or driving factor in various diseases (Du et al., 2023; Jiang et al., 2019). For instance, gastrointestinal-related diseases such as diarrhea, intestinal cancer, inflammatory bowel disease, and constipation are often accompanied by gut microbial dysbiosis (Du et al., 2023; Zhuang et al., 2021). Additionally, gut microbial dysbiosis is also considered to be one of the key factors in the occurrence and progression of obesity, diabetes, and hypertension, possibly through pro-inflammatory responses and disruption of intestinal metabolism (de Clercq et al., 2016; Hasain et al., 2020). Gut microbial dysbiosis not only affects intestinal function but can also have systemic negative effects. Therefore, it is crucial to maintain the gut microbial balance to ensure host health and proper intestinal function.

Horses are non-ruminant, odd-toed ungulates, and monogastric herbivorous mammals that have played a significant role in human civilization and social development (Jin et al., 2023). Early investigations have indicated that horses are the oldest domesticated animals, dating back to about 5,500 B.C. Throughout history, humans have selectively bred horses based on social needs, such as appearance, strength, speed, and tolerance, resulting in variations in traits among different horse breeds (Mach et al., 2017). Currently, there are approximately 59 million horses worldwide, encompassing 300 different breeds, with an estimated annual economic impact of around US$300 billion. As a domesticated species vital to humans, horses are bred and utilized globally for purposes such as racing, entertainment, transportation, agricultural production, as well as being important sources of meat and milk in developing countries (Stanislawczyk et al., 2021). In recent years, there has been an increasing demand to selectively breed horses with desirable phenotypic, morphological, and functional characteristics for success in equestrian competition. However, the process of domestication and modern breeding practices have led to a significant reduction in genetic diversity and the accumulation of harmful genetic variations within the equine genome. For instance, domesticated horses exhibit reduced microbial diversity in their gut microbiota compared to wild horses. Therefore, it is crucial to comprehend the biology of horses in order to ensure their well-being and enhance their utilization in human activities.

Presently, high-throughput sequencing technology has been widely used to explore the differences in gut microbiota among different species (Wei et al., 2021). For instance, Massacci et al. (2020) found that the gut microbial diversity and abundance of Hanoverian horses were significantly higher than those of Lusitano horses. Similarly, Park et al. (2021) observed that the gut microbial diversity and the beneficial bacteria producing short-chain fatty acids are significantly higher in Thoroughbred horses compared to Jeju horses. The GZH, a local breed found in southwest China, is known for its short body, delicate appearance, agile movement, and docility. Meanwhile, WH are specifically bred for equestrian competition and possess a range of exceptional characteristics (Wijnberg et al., 2003). Previous research has indicated that the traits of different horse breeds are closely related to gut microbiota in addition to genes. However, there are currently no studies available that explore the gut microbiota of WH and GZH. In this study, we conducted a comparative analysis to examine the differences in the gut bacterial and fungal communities between these two horse breeds.

## Materials and methods

### Sample collection

A total of 8 GZH (about 5 years old) and 8 WH (about 5 years old) were chosen as subjects for this study. Horses within the same group share identical diet and housing environments. Each group consisted of four male horses and four female horses. Health evaluations were performed prior to sampling to minimize the influence of other variables on the gut microbiota. Furthermore, none of the sampled horses had received prior antibiotic injections. Fresh fecal samples were obtained from each horse’s rectum using a fecal sampler and stored at −80°C for further analysis.

### 16S rDNA amplicon sequencing

For each selected sample from different treatment groups, DNA extraction was performed using commercial kits following the manufacturer’s instructions. The integrity, concentration, and purity of the extracted DNA were tested based on previous studies to ensure that its quality met the requirements for subsequent analysis (Hubert et al., 2019; Park et al., 2021). Additionally, universal primers (338F: ACTCCTACGGGAGGCAGCA and 806R: GGACTACHVGG GTWTCTAAT; ITS5F: GGAAG TAAAAGTCGTAACAAGG and ITS2R: GCTGCGTTCTTCATCGA TGC) were synthesized to amplify the V3/V4 and ITS2 regions. PCR amplification was carried out in triplicate with 20 μL volumes, following the amplification conditions described in previous studies (Guan et al., 2023). The quality of the PCR amplification products was assessed, and the target fragments were then recovered. To prepare sequencing libraries, the recovered products underwent further purification and quantification. The prepared library underwent a series of evaluations, including quality inspection and quantification, to determine its eligibility. Libraries with concentrations greater than 2 nM, no adapters, and only a single peak were considered qualified. The final qualified library was subjected to 2 × 300 bp paired-end sequencing on the MiSeq sequencer.

### Bioinformatics and statistical analysis

Some problematic sequences contained in the original sequence include chimeras, low-quality, and short sequences that need to be eliminated to obtain qualified sequences. Specifically, the initial reads produced by amplicon sequencing were first filtered using Trimmomatic v0.33 software. Subsequently, the cutadapt 1.9.1 software was used for identifying and removing the primer sequences to obtain clean reads. Moreover, rarefaction curves and rank abundance curves were generated for each sample in different treatment groups to assess sequencing depth and evenness. High-quality sequences in each sample were clustered into OTUs at 97% similarity. The number of OTUs in different groups or samples was displayed using a Venn diagram. Additionally, we plotted the composition and abundance map of the gut microbial community at different taxonomic levels based on the OTUs analysis results. Microbial alpha diversity indices, such as Chao1, ACE, Shannon, and Simpson, were computed using the number of OTUs in each sample to assess the diversity and abundance of gut microbiota. Beta diversity analysis was also performed to assess changes in the gut microbial structure, and the results were visualized using PCoA scatterplots. Taxa with statistical differences between different treatment groups were identified using Metastats analysis and Lefse analysis. Statistical analysis of data was performed using R (v3.0.3) and GraphPad Prism (version 9.0c). The data were expressed as mean ± SEM, and statistical significance was considered at *p* < 0.05.

## Results

### Analysis of sequence data and OTUs numbers

**Figure 1:**
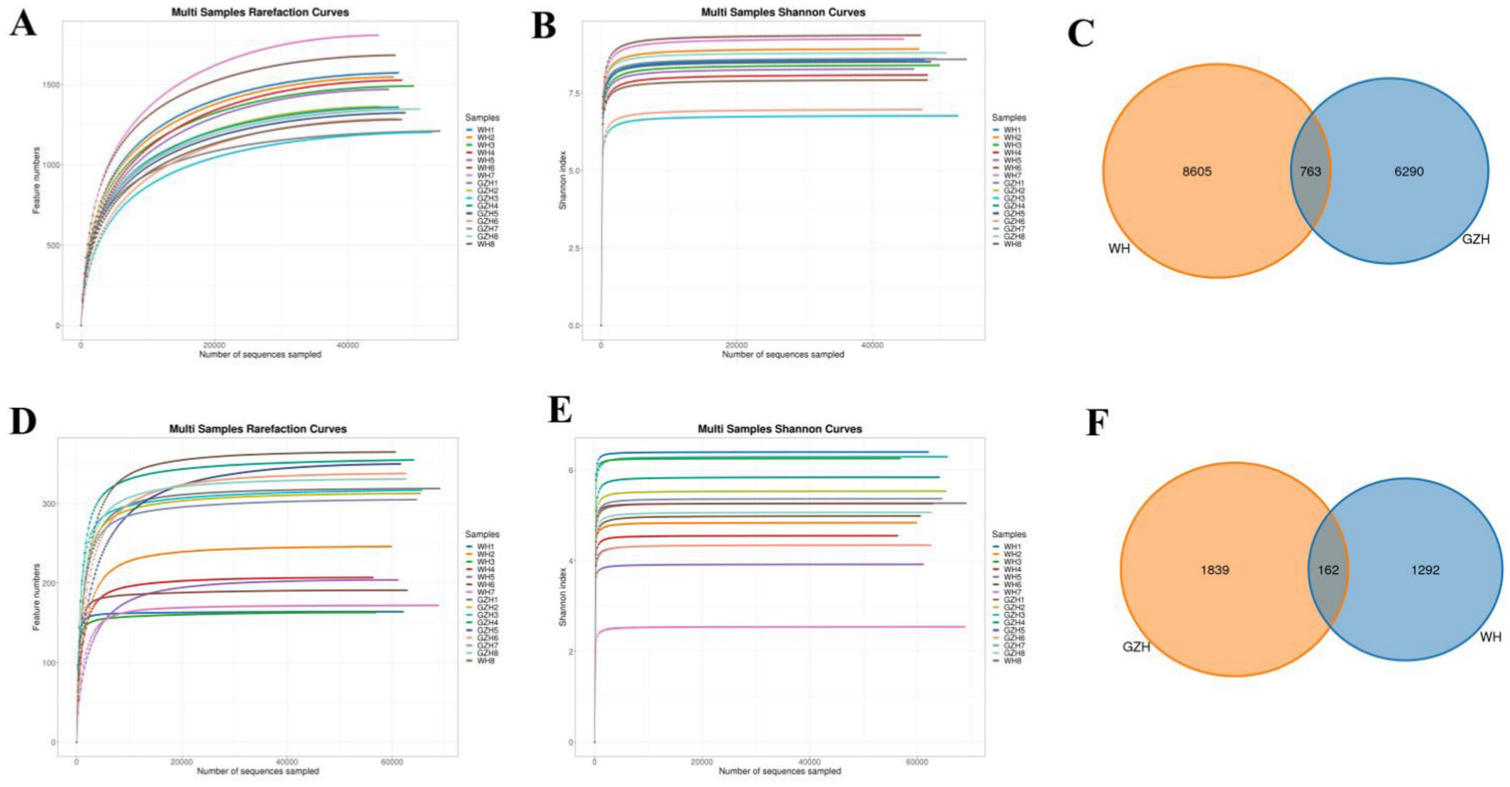
OTUs distribution and sequencing data analysis. Gut bacterial **(A,B)** and fungal **(D,E)** rarefaction curves evaluate sequencing depth and uniformity. Venn diagram showing the number of shared or individual OTUs of gut gut bacterial **(C)** and fungal **(F)** communities in GZH and WH.

To compare the gut bacterial and fungal communities between GZH and WH, amplicon sequencing was conducted on fecal samples. A total of 1,279,446 original bacterial sequences (Table 1) and 1,279,352 fungal sequences (Table 2) were initially obtained. We also further screened and filtered these raw sequences to obtain valid sequences. Results indicated that 781,011 effective bacterial sequences and 1,004,642 effective fungal sequences were identified, with both exceeding 61 and 78% effectiveness, respectively. Rarefaction curves were utilized to evaluate sequencing depth and uniformity. The findings indicated that further increasing sequencing depth does not lead to the discovery of additional bacterial (Figures 1A,B) and fungal taxa (Figures 1D,E), suggesting that the current sequencing depth and uniformity are adequate. Subsequent clustering of valid sequences resulted in 15,658 bacterial OTUs (Figure 1C) and 3,293 fungal OTUs (Figure 1F). Notably, 763 bacterial OTUs and 162 fungal OTUs were shared between GZH and WH. Moreover, GZH exhibited 6,290 individual bacterial OTUs and 1,839 individual fungal OTUs. In contrast, WH displayed 8,605 individual bacterial OTUs and 1,292 individual fungal OTUs.

**Table 1:** Statistics of bacterial raw and valid sequences produced by amplicon sequencing.

Sample ID	Raw reads	Clean reads	Denoised reads	Merged reads	Non-chimeric reads
WH1	80,049	72,226	71,596	60,767	48,164
WH2	80,012	72,030	71,470	59,825	47,365
WH3	80,058	71,904	71,393	60,998	50,333
WH4	79,918	71,640	71,092	59,416	48,590
WH5	80,006	71,829	71,348	59,759	46,623
WH6	80,056	72,136	71,460	59,479	47,656
WH7	79,909	72,163	71,558	59,363	45,136
WH8	79,858	72,100	71,675	61,335	48,499
GZH1	80,032	73,398	72,959	63,879	54,237
GZH2	80,032	73,502	72,925	60,427	45,406
GZH3	79,921	72,367	71,912	64,273	53,081
GZH4	79,944	71,947	71,431	59,964	47,941
GZH5	80,045	72,774	72,298	61,130	49,023
GZH6	79,921	72,128	71,687	61,670	47,900
GZH7	79,946	72,611	72,126	61,416	49,836
GZH8	79,739	72,472	71,918	61,585	51,221

**Table 2:** Statistics of fungal raw and valid sequences produced by amplicon sequencing.

Sample ID	Raw reads	Clean reads	Denoised reads	Merged reads	Non-chimeric reads
WH1	80,022	66,502	66,402	65,839	62,213
WH2	79,632	64,739	64,707	60,455	59,941
WH3	79,933	63,662	63,607	63,176	56,852
WH4	79,881	59,713	59,662	58,622	56,441
WH5	80,059	63,874	63,860	62,055	61,125
WH6	79,964	64,658	64,527	63,920	62,787
WH7	80,024	69,495	69,488	68,924	68,837
WH8	79,939	63,872	63,816	62,370	60,685
GZH1	80,109	70,071	69,974	69,512	69,141
GZH2	79,990	67,339	67,318	66,461	65,474
GZH3	80,000	68,143	68,104	67,336	65,631
GZH4	79,809	68,139	68,056	66,990	64,028
GZH5	79,984	63,770	63,750	62,883	61,613
GZH6	80,157	63,348	63,330	62,699	62,592
GZH7	79,677	65,981	65,957	65,399	64,618
GZH8	80,172	63,987	63,965	63,192	62,664

### Differences in gut bacterial and fungal diversities index

**Figure 2:**
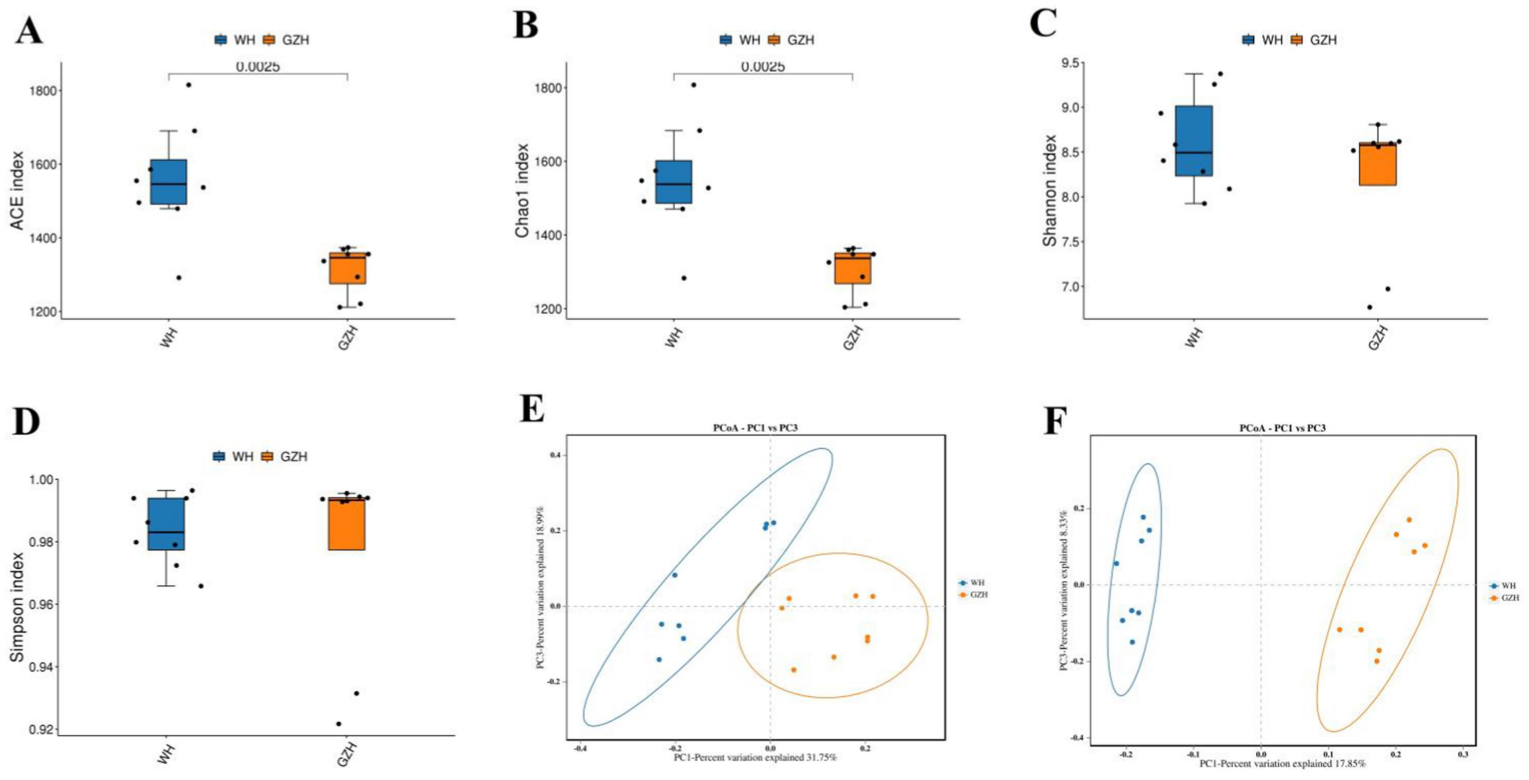
Boxplots showing the gut bacterial diversity measured as the ACE **(A)**, Chao1 **(B)**, Shannon **(C)**, and Simpson **(D)** in the GZH and WH. Differences in gut bacterial structure between the GZH and WH were evaluated by PCoA scatter plots **(E,F)**. All the data represent means ± SD. *p*-values <0.05 were considered statistically significant.

**Figure 3:**
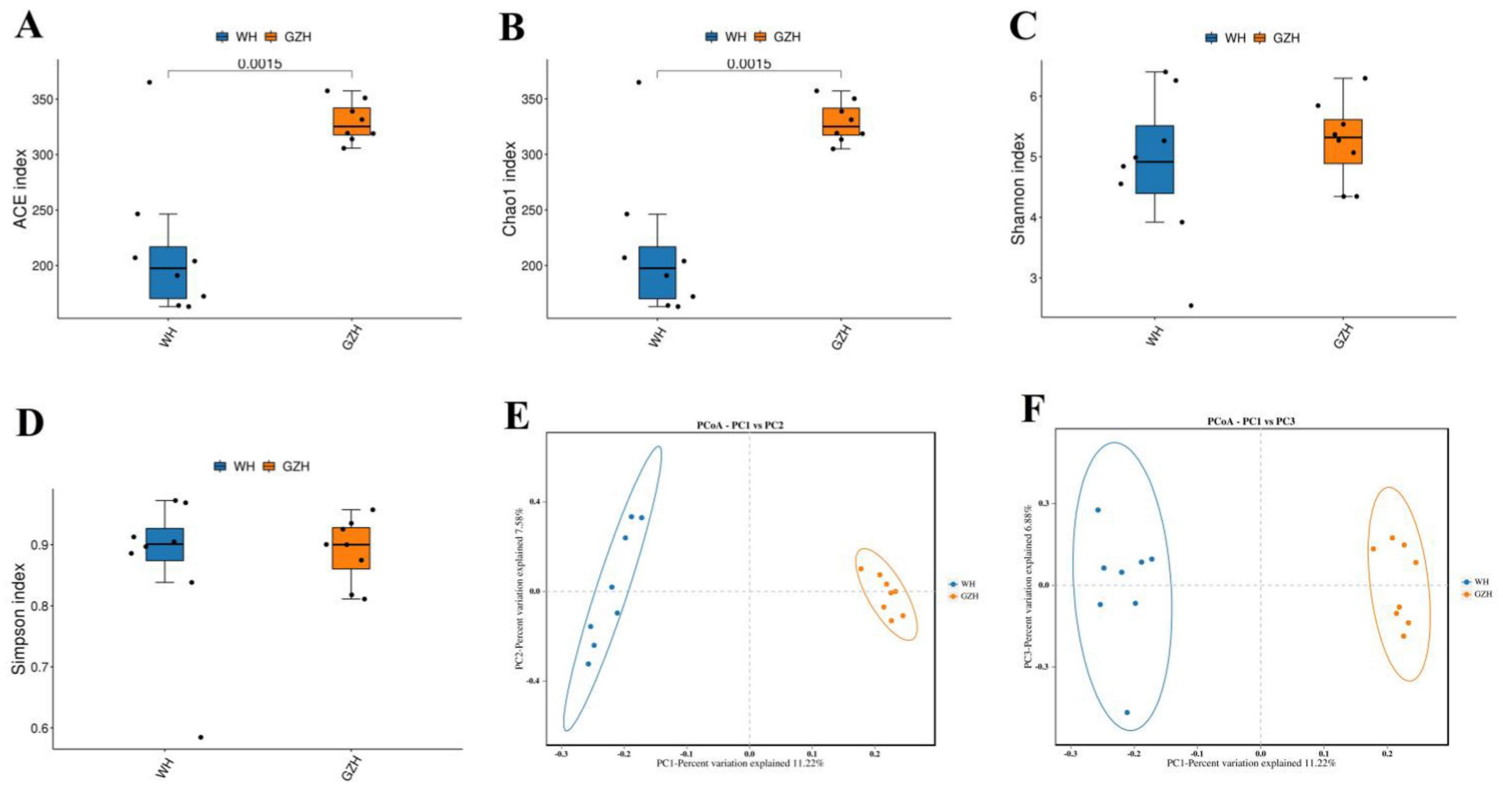
Boxplots showing the gut fungal diversity measured as the ACE **(A)**, Chao1 **(B)**, Shannon **(C)** and Simpson **(D)** in the GZH and WH. Differences in gut fungal structure between the GZH and WH were evaluated by PCoA scatter plots **(E,F)**. All the data represent means ± SD. *p*-values <0.05 were considered statistically significant.

The Chao1, ACE, Simpson, and Shannon indices of the gut bacterial community were determined for GZH and WH. For GZH, the indices were 1305.97, 1314.47, 0.97, and 8.17 (Figures 2A–D), while for WH, the corresponding values were 1548.18, 1556.15, 0.98 and 8.60 (Figures 3A–D). Comparative analysis revealed that the Chao1 and ACE indices of WH were significantly greater than those of GZH, indicating a higher abundance of gut bacterial community in WH. In contrast, the Simpson and Shannon indices did not show significant differences between the two groups, suggesting similar levels of bacterial diversity in both WH and GZH. The Chao1, ACE, Simpson, and Shannon indices of the gut fungal community in GZH were 329.18, 329.63, 0.89, and 5.25, respectively. Conversely, the four diversity indices of the gut fungal community in WH were 214.02, 214.12, 0.87, and 4.84, respectively. Analysis of the gut fungal community revealed that the Chao1 and ACE indices in GZH were significantly higher than those in WH, while the Simpson and Shannon indices did not show a significant difference. This suggests that the abundance of the gut fungal community in GZH was notably higher than in WH, but there was no disparity in fungal diversity between the two groups. To further investigate the changes in gut bacterial and fungal communities between GZH and WH, we conducted a comparative analysis of their structures using PCoA. The PCoA analysis revealed distinct separation between the data points representing GZH and WH, suggesting significant differences in the composition of gut bacterial community (Figures 2E,F). Similarly, the analysis of gut fungal community also demonstrated notable divergence between the two groups (Figures 3E,F).

### Gut bacterial compositions and taxonomic differences between GZH and WH

**Figure 4:**
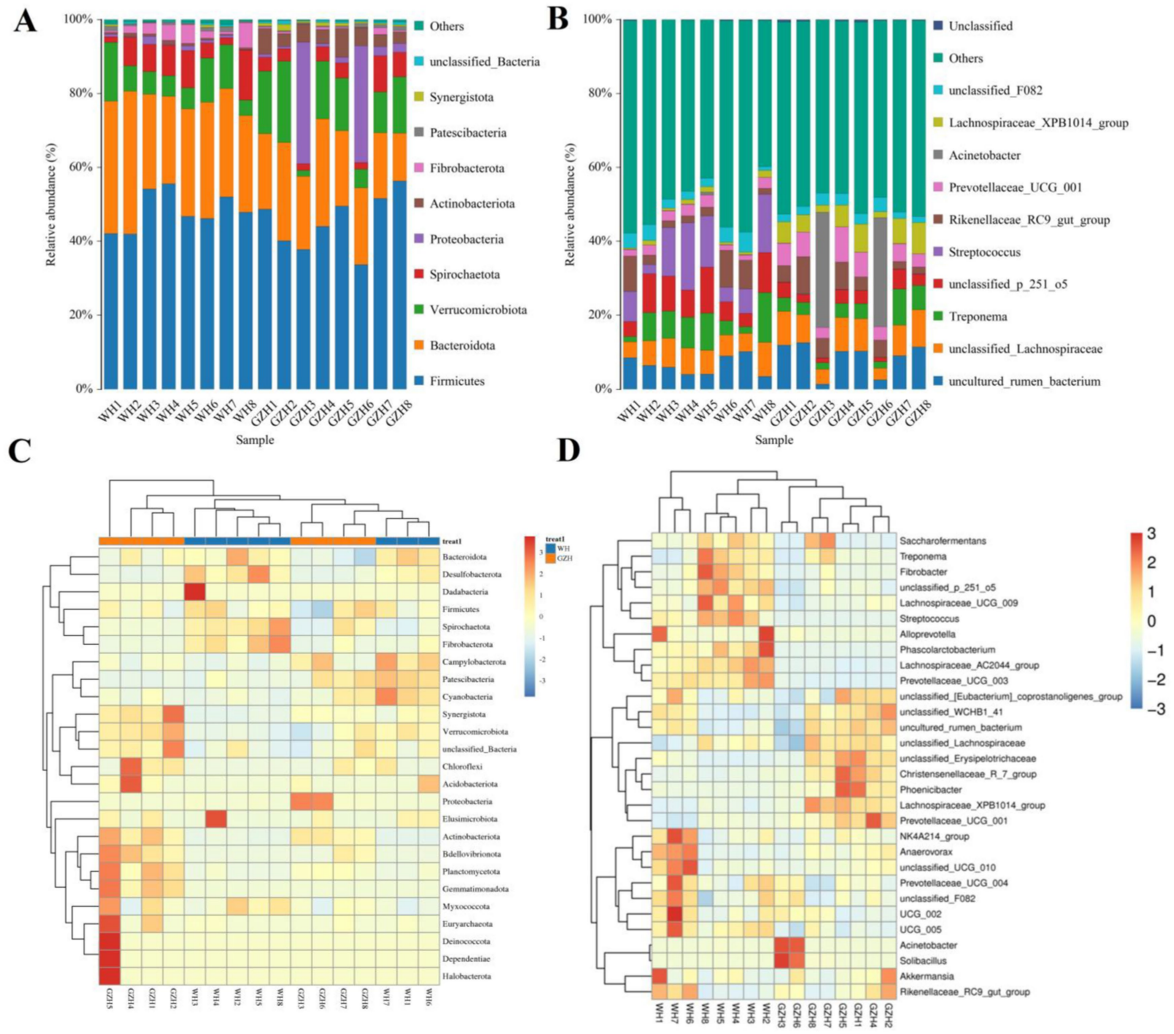
Changes in the relative abundances of bacteria at phylum **(A)** and genus **(B)** levels in GZH and WH. The clustering heatmap show the relative proportion and distribution of more bacterial phylum **(C)** and genus **(D)**.

In this study, a total of 25 bacterial phyla and 464 bacterial genera were identified from GZH and WH. At the phylum level, the *Firmicutes* (45.35, 48.33%), *Bacteroidota* (20.82, 29.93%), and *Verrucomicrobiota* (12.60, 8.47%) were the predominant phyla in the GZH and WH (Figure 4A). At the genus level, the *Streptococcus* (10.34%) was the most predominant bacterial genus in the WH, followed by *unclassified_p_251_o5* (7.95%), *Treponema* (6.77%), and *unclassified_Lachnospiraceae* (6.50%). Moreover, *uncultured_rumen_bacterium* (8.61%), *Acinetobacter* (7.71%), *unclassified_Lachnospiraceae* (7.52%) and *Lachnospiraceae_XPB1014_group* (5.35%) were abundantly present in the GZH (Figure 4B). Besides the above dominant bacterial phyla and genera, other bacterial abundance was also analyzed and visualized by clustering heatmaps (Figures 4C,D).

**Figure 5:**
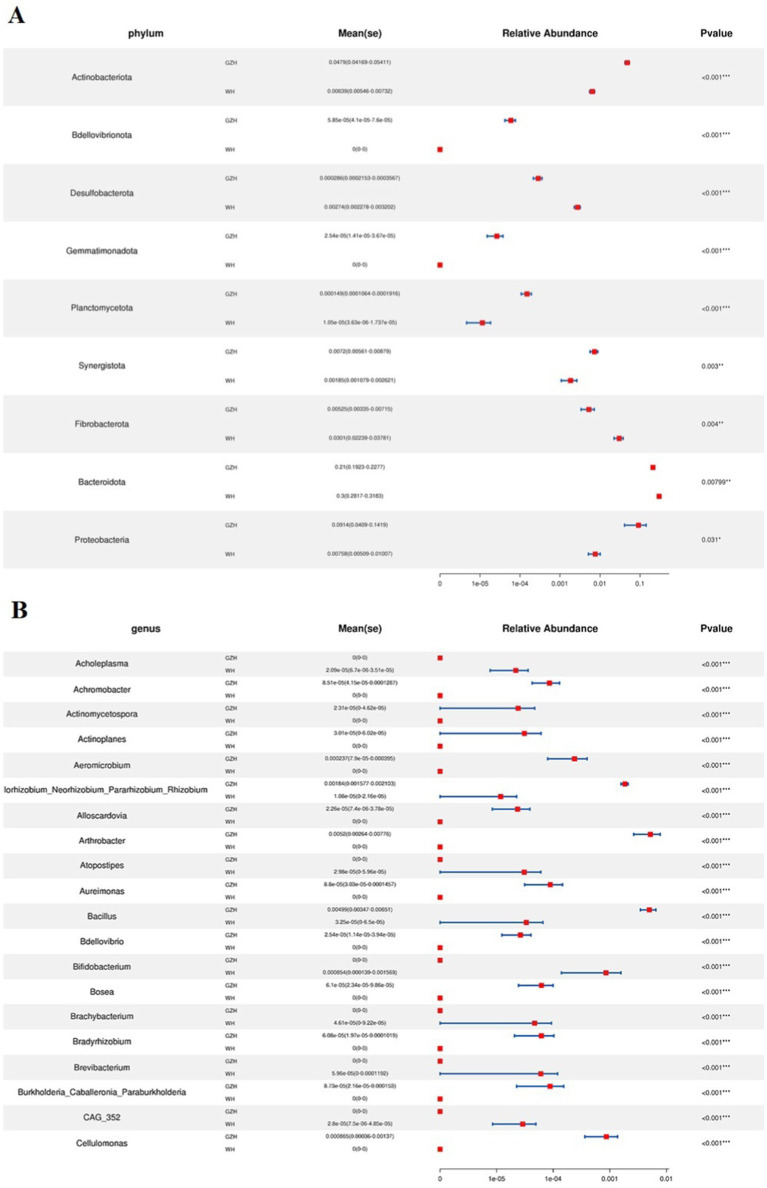
Comparison of gut bacterial community at the phylum **(A)** and genus **(B)** level between GZH and WH. Data were not fully shown. All the data represent means ± SD. *p < 0.05, **p < 0.01, ***p < 0.001.

**Figure 6:**
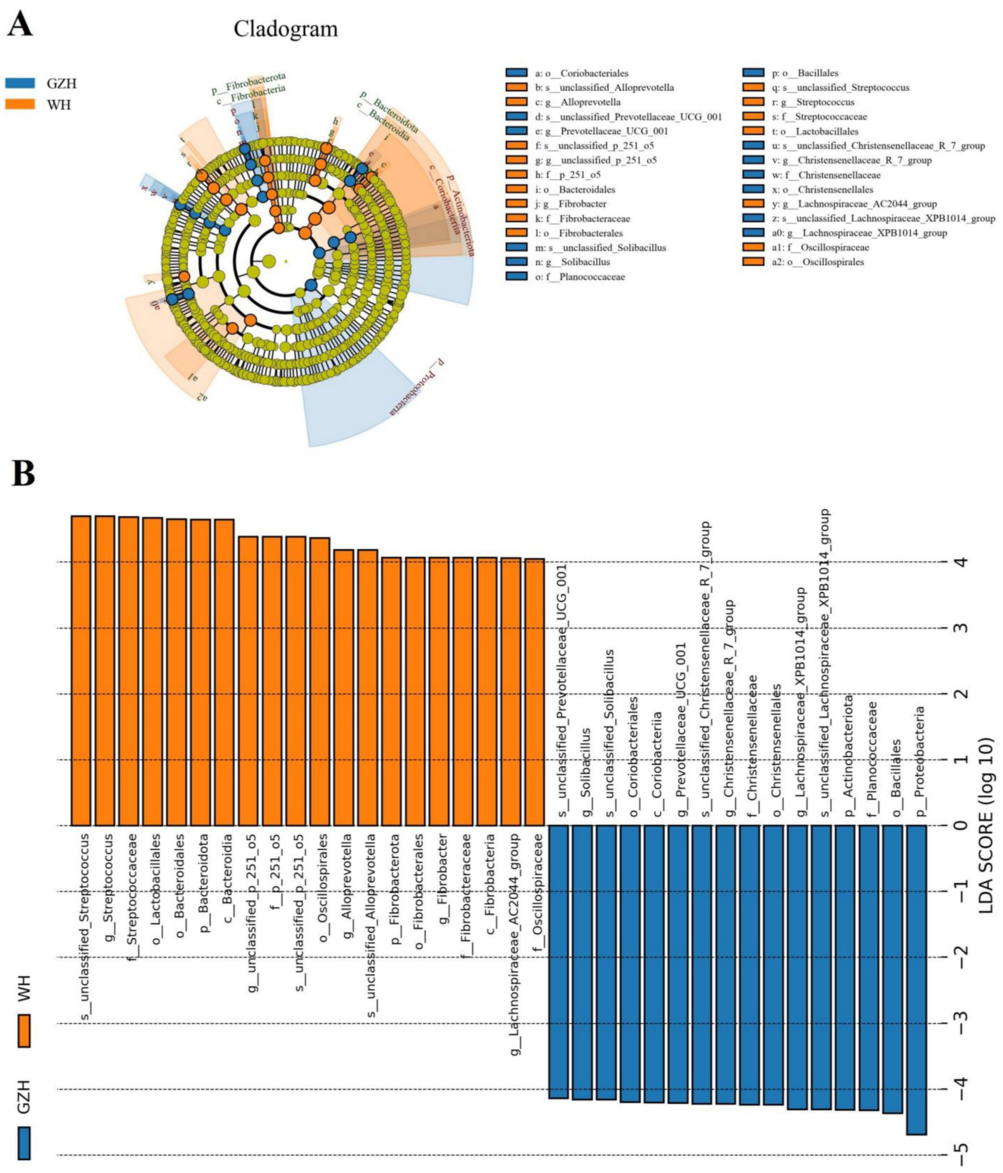
Differential bacteria at different taxonomic levels related to GZH or WH. **(A)** Cladogram. **(B)** LDA scores. Only LDA values >4 can be displayed.

To investigate the difference in gut bacterial community between GZH and WH, we conducted Metastats analysis and LEfSe analysis to identify differential taxa at the phylum and genus levels. At the phylum level, the abundances of *Actinobacteriota*, *Bdellovibrionota*, *Gemmatimonadota*, *Planctomycetota*, *Synergistota* and *Proteobacteria* in the GZH were significantly dominant than WH, while the *Desulfobacterota*, *Fibrobacterota* and *Bacteroidota* were lower (Figure 5A). Furthermore, 217 bacterial genera were significantly different between GZH and WH. Specifically, the abundances of 123 bacterial genera (*Achromobacter*, *Actinomycetospora*, *Actinoplanes*, *Aeromicrobium*, *Bacillus*, *Bdellovibrio*, *Bifidobacterium*, *Christensenellaceae_R_7_group*, *Lachnospiraceae_XPB1014_group*, *Lactococcus*, *Limosilactobacillus*, *Prevotella_7*, *Prevotellaceae_UCG_001*, *Ruminiclostridium*, etc.) in GZH was significantly higher than that in WH, while the abundances of 94 bacterial genera (*Lachnospiraceae_AC2044_group*, *Oscillospira*, *Phascolarctobacterium*, *Prevotellaceae_UCG_003*, *Pygmaiobacter*, *Ruminococcus*, *Weissella*, *Anaeroplasma*, *Papillibacter*, *Lachnospiraceae_UCG_009*, *Pseudobutyrivibrio*, etc.) was significantly lower than that in WH (Figure 5B). Interestingly, we observed that 92 bacterial genera (*Achromobacter*, *Actinomycetospora*, *Actinoplanes*, *Aeromicrobium*, *Alloscardovia*, *Bdellovibrio*, *Chryseobacterium*, *Curtobacterium*, *Diplorickettsia*, *Prevotella_7*, *Lactococcus*, etc.) were only present in GZH, while 46 bacterial genera (*Acholeplasma*, *Atopostipes*, *Bifidobacterium*, *Brachybacterium*, *Brevibacterium*, *Corynebacterium*, *Dubosiella*, *Kurthia*, *Lachnoclostridium*, *Lachnospiraceae_NK4B4_group*, *Lachnospiraceae_UCG_007*, etc.) were only present in WH. Moreover, LEfSe analysis results showed that the *unclassified_Erysipelotrichaceae* and *Solibacillus* in the gut bacterial community of GZH were significantly higher than those of WH, while the abundances of *Streptococcus*, *unclassified_p_251_o5*, and *Alloprevotella* was lower (Figures 6A,B).

### Gut fungal compositions and taxonomic differences between GZH and WH

**Figure 7:**
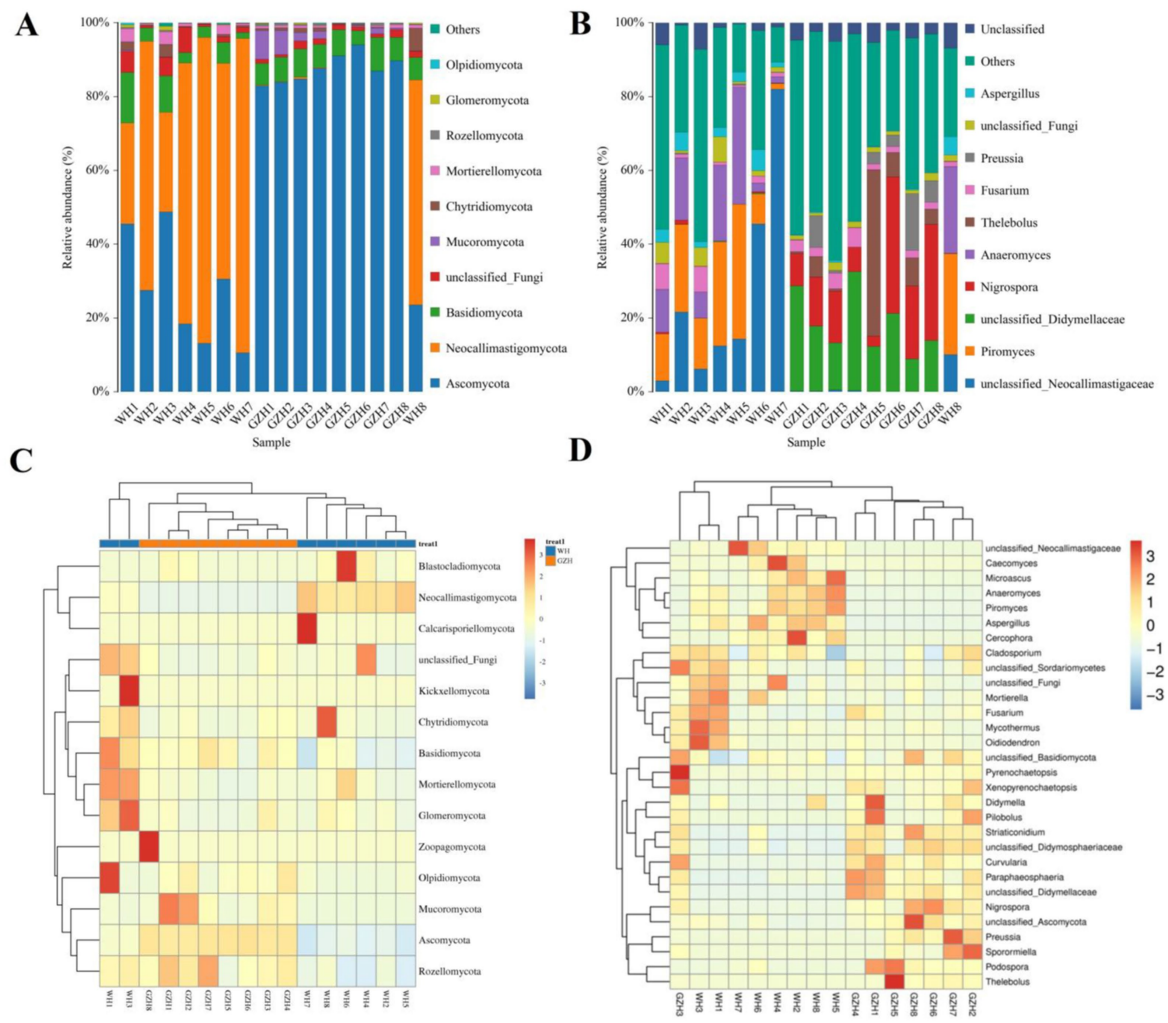
Changes in the relative abundances of fungi at phylum **(A)** and genus **(B)** levels in GZH and WH. The clustering heatmap show the relative proportion and distribution of more fungal phylum **(C)** and genus **(D)**.

There were 14 fungal phyla and 563 fungal genera found in the gut fungal community of GZH and WH. At the phylum level, the average abundances of 6 fungal phyla including *Ascomycota* (26.92%), *Neocallimastigomycota* (60.50%), *Basidiomycota* (5.74%), *unclassified_Fungi* (2.84%), *Chytridiomycota* (1.68%), and *Mortierellomycota* (1.50%) in the gut fungal community of WH exceeded 1% (Figure 7A). Moreover, the *Ascomycota* (87.52), *Basidiomycota* (6.59%), *unclassified_Fungi* (1.38%) and *Mucoromycota* (2.68%) were the most dominant phyla, with an average abundance exceeding 1%. At the genus level, the dominant fungi found in WH were *unclassified_Neocallimastigaceae* (25.60%), *Piromyces* (18.58%), *Anaeromyces* (14.18%), *unclassified_Fungi* (2.84%) and *Aspergillus* (3.41%) (Figure 7B). Moreover, 6 abundant fungi such as *unclassified_Didymellaceae* (18.55%), *Nigrospora* (16.57%), *Thelebolus* (8.52), *Fusarium* (2.77%), *Preussia* (4.60%), and *unclassified_Fungi* (1.38%) which were defined as containing over 1% in the gut fungal community of GZH. Heatmaps are useful tools for visualizing the abundance and diversity of fungal phyla and genera, enabling researchers to observe changes in these taxonomic groups (Figures 7C,D).

**Figure 8:**
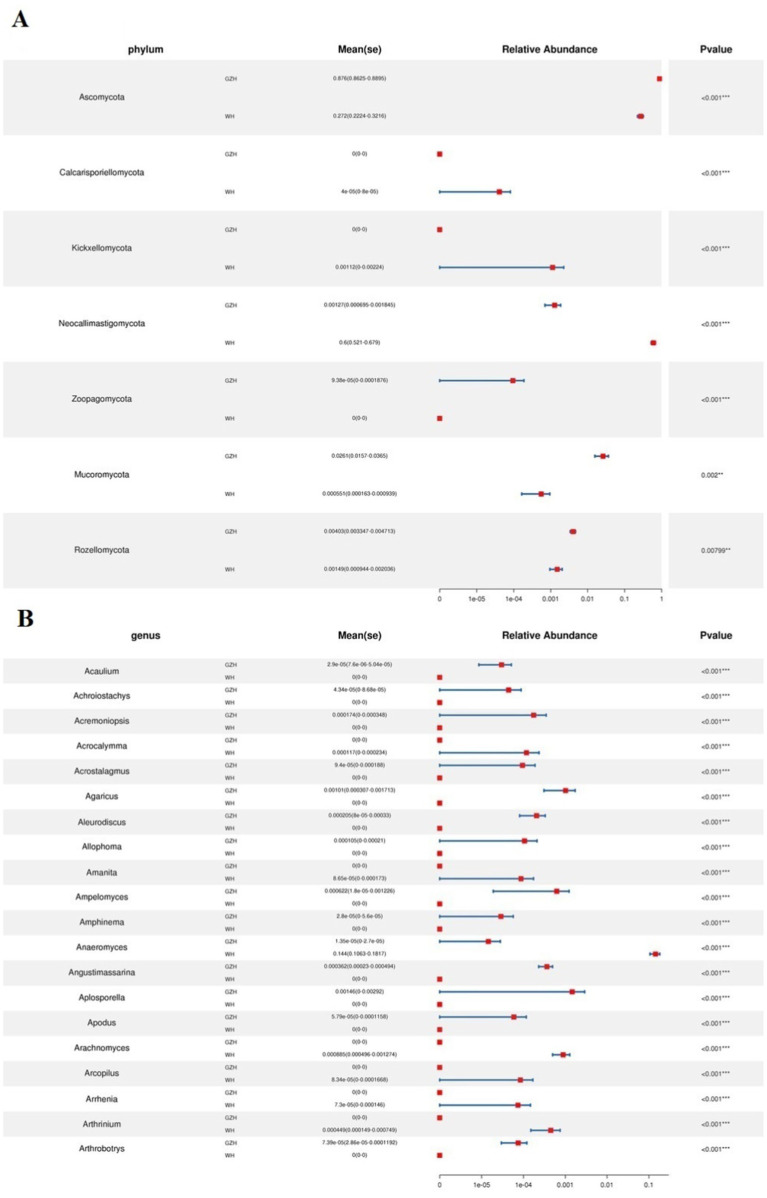
Comparison of gut fungal community at the phylum **(A)** and genus **(B)** level between GZH and WH. Data were not fully shown. Data were not fully shown. All the data represent means ± SD. *p < 0.05, **p < 0.01, ***p < 0.001.

**Figure 9:**
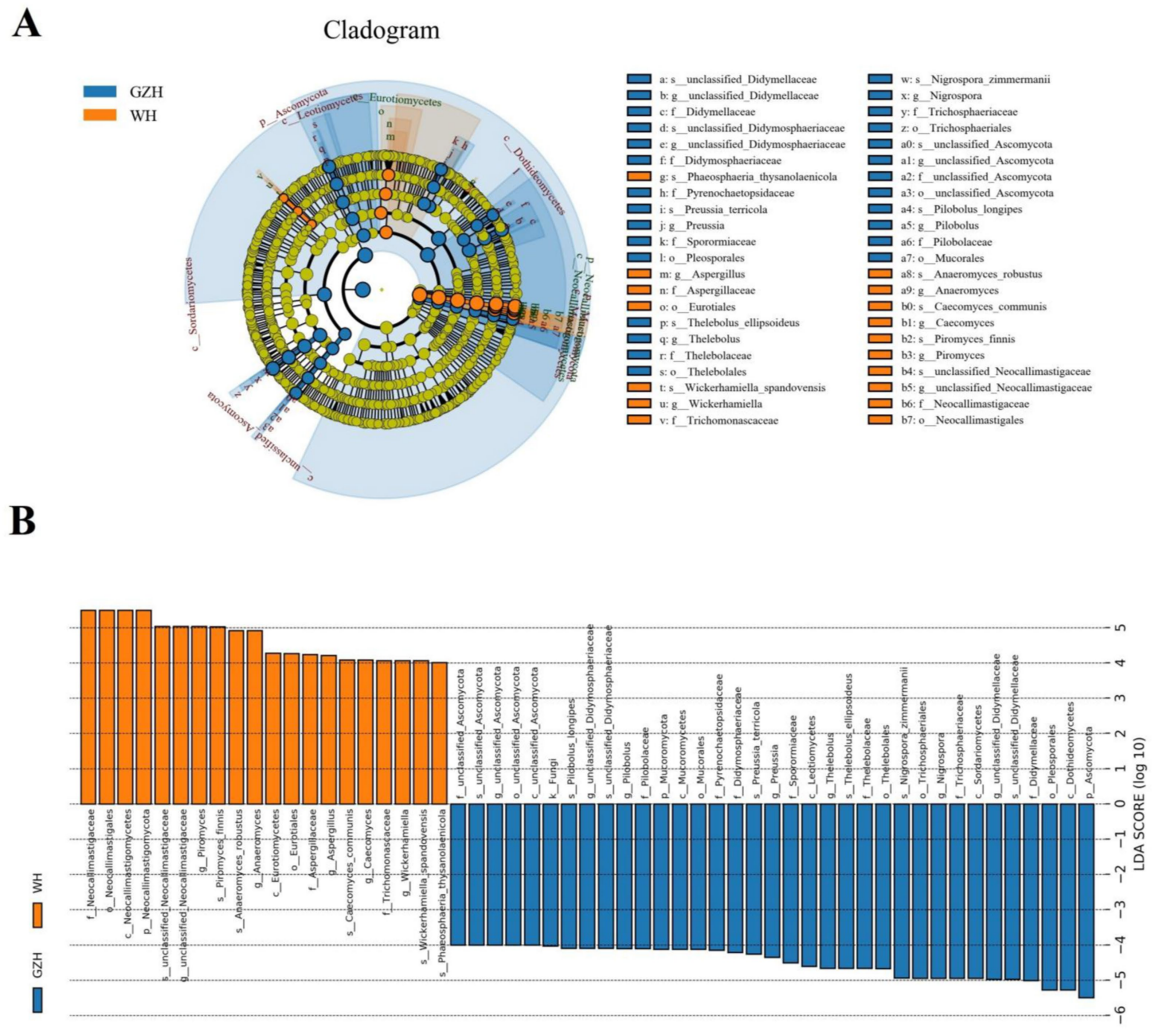
Differential fungi at different taxonomic levels related to GZH or Dutch WH. **(A)** Cladogram. **(B)** LDA scores. Only LDA values >4 can be displayed.

At the phylum level, the abundances of *Zoopagomycota*, *Mucoromycota*, *Rozellomycota*, and *Ascomycota* in GZH was significantly higher than in WH, whereas the abundances of *Calcarisporiellomycota*, *Kickxellomycota*, and *Neocallimastigomycota* was lower (Figure 8A). Moreover, a total of 365 fungal genera exhibited significant differences between GZH and WH. Specifically, the abundances of 229 fungal genera (*Cystobasidium*, *Nigrospora*, *Striaticonidium*, *Ustilago*, *Xenopyrenochaetopsis*, *Myxospora*, *Nemania*, *Neoascochyta*, etc.) in GZH was significantly higher than in WH, while 136 fungal genera (*Anaeromyces*, *Aspergillus*, *Hohenbuehelia*, *Hortaea*, *Hymenula*, *Lecanicillium*, *Leucoagaricus*, *Leucocoprinus*, *Lophiotrema*, etc.) had significantly lower abundance in GZH compared to WH (Figure 8B). Additionally, 127 fungal genera (*Acrocalymma*, *Amanita*, *Arachnomyces*, *Arcopilus*, *Arrhenia*, *Arthrinium*, *Arthrocatena*, *Arxiella*, *Ascochyta*, *Ascotricha*, *Beauveria*, *Botryosphaeria*, *Caecomyces*, *Calcarisporiella*, *Calycina*, ect.) were completely absent in the gut fungal community of GZH. Similarly, 201 fungal genera (*Acaulium*, *Achroiostachys*, *Acremoniopsis*, *Acrostalagmus*, *Agaricus*, *Aleurodiscus*, *Allophoma*, *Ampelomyces*, *Angustimassarina*, *Aplosporella*, *Apodus*, *Arxotrichum*, *Ascobolus*, etc.) were undetectable in the gut fungal community of WH. The LEfSe analysis and LDA scores were utilized to further elucidate the differences between GZH and WH (Figures 9A,B).

### Correlation network analysis

**Figure 10:**
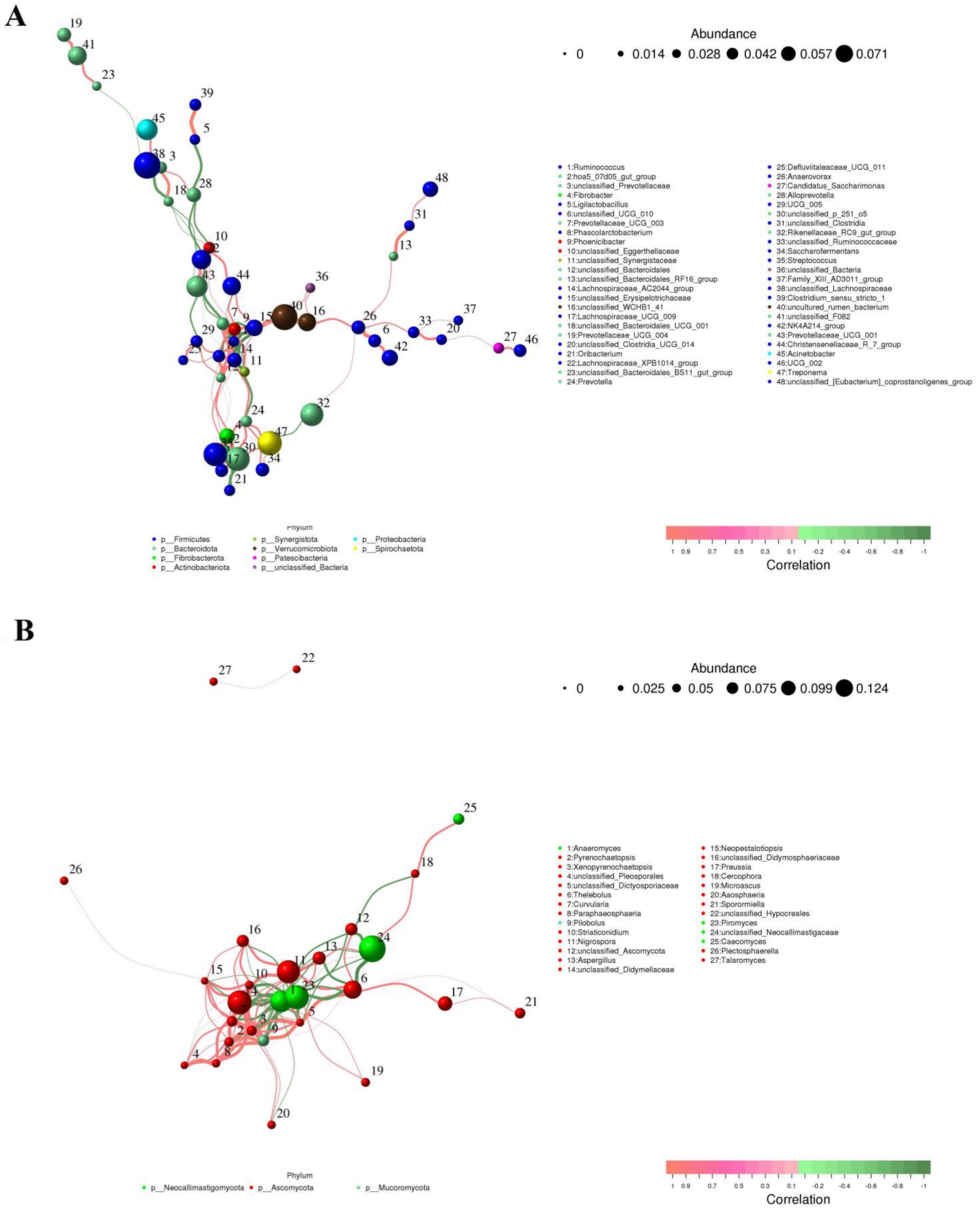
Co-occurrence networks of bacterial **(A)** or fungal **(B)** genera constructed on the WH and GZH. The red line between the both genera represents a positive correlation, whereas the green line indicates a negative correlation.

Representative gut bacterial and fungal communities were analyzed using Python to investigate correlations between them. Figure 10 illustrates the direct relationship between select bacteria and fungi. In the gut bacterial community, *Ruminococcus* showed a positive association with *Lachnospiraceae_AC2044_group*, *Prevotellaceae_UCG_003* (0.87) and *Phascolarctobacterium* (0.86). However, it was inversely related to *Christensenellaceae_R_7_group* (−0.75), *Phoenicibacter* (−0.89), and *unclassified_Erysipelotrichaceae* (−0.88). *Lachnospiraceae_UCG_009* was negatively correlated with *Fibrobacter* (0.85), *Streptococcus* (0.80) and *unclassified_p_251_o5* (0.80). *Prevotella* was positively associated with *unclassified_p_251_o5* (0.81), *Fibrobacter* (0.81), *Treponema* (0.79), *Lachnospiraceae_AC2044_group* (0.79), *Saccharofermentans* (0.78) and *Saccharofermentans* (0.78). *Phascolarctobacterium* exhibited positive associations with *Lachnospiraceae_AC2044_group* (0.89), *Prevotellaceae_UCG_003* (0.79), *Fibrobacter* (0.76), and *Streptococcus* (0.75), while showing an inverse relationship with *Phoenicibacter* (−0.84).

In the gut fungal community, *Xenopyrenochaetopsis* showed a positive correlation with *Pilobolus* (0.95), *Curvularia* (0.86), *unclassified_Didymellaceae* (0.83), and *Didymella* (0.79), but had an inverse relationship with *Anaeromyces* (−0.85) and *Piromyces* (−0.83). *Neopestalotiopsis* exhibited a positive correlation with *Xenopyrenochaetopsis* (0.92), *Aaosphaeria* (0.91), *Pilobolus* (0.87), *Striaticonidium* (0.86), *Curvularia* (0.84), *unclassified_Didymellaceae* (0.83), *unclassified_Dictyosporiaceae* (0.84), *Nigrospora* (0.82), *Preussia* (0.78), *Paraphaeosphaeria* (0.78), and *Thelebolus* (0.77), while being inversely related to *Anaeromyces* (−0.85) and *Piromyces* (−0.85). *Aaosphaeria* was positively correlated with *Xenopyrenochaetopsis* (0.90), *Pilobolus* (0.90), *unclassified_Didymellaceae* (0.83), *Paraphaeosphaeria* (0.79), *Thelebolus* (0.79), and *Striaticonidium* (0.78), but had inverse relationships with *Anaeromyces* (−0.85), *Piromyces* (−0.85), and *Aspergillus* (−0.80).

### Functional analysis of gut microbiota

**Figure 11:**
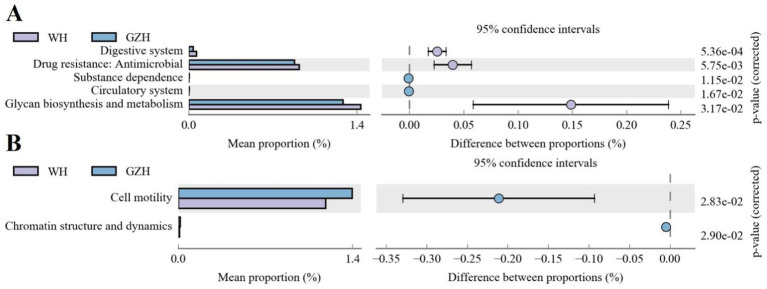
Functional predictive analysis of gut bacterial community. **(A)** KEGG metabolic pathway histogram. **(B)** COG metabolic pathway histogram. All the data represent means ± SD. *p*-values <0.05 were considered statistically significant.

PICRUSt software was utilized to compare the composition of gut microbiota and analyze the functional gene composition and differences between GZH and WH. Gut bacterial KEGG functional prediction analysis showed that the relative abundances of glycan biosynthesis and metabolism, drug resistance: antimicrobial and digestive system were significantly increased in WH, while the substance dependence and circulatory system were decreased as compared to GZH (Figure 11A). In the COG functional prediction analysis, the abundance of cell motility and chromatin structure and dynamics in the GZH was significantly higher than that in WH (Figure 11B).

## Discussion

The composition and diversity of gut microbiota are crucial for maintaining intestinal homeostasis and host health (Zhang Z. et al., 2022). Horses, like many other species, harbor a complex gut microbiota that significantly impacts their growth, metabolism, immunity, and digestion (Li et al., 2022; Mach et al., 2020). The gut microbiota of horses has evolved into a delicate balance with the host and the environment as the host has evolved (Di Pietro et al., 2021). However, this balance can be easily influenced by external factors such as dietary structure, environmental conditions, and age (Tizabi et al., 2023; Zhang et al., 2023). Recent studies on various varieties of the same species have also shown significant effects of variety on gut microbiota. For example, Ma et al. (2022) demonstrated significant changes in the gut microbiota of Duroc, Landrace and Yorkshire pigs. Moreover, significant differences in the composition and structure of gut microbiota have also been observed between Kasaragod Dwarf and Holstein crossbred cattle (Deepthi et al., 2023). China possesses a large population of horses and abundant horse breed resources (Yang et al., 2017). GZH are significant horse breeds in China that play a crucial role in local economic development and the livelihoods of residents. Despite this, there has been limited analysis conducted on the comparative study of the gut microbiota of WH and GZH. To address this gap, we collected fecal samples from WH and GZH and conducted 16S rDNA sequencing to investigate the composition and discrepancies in gut microbiota between these two breeds.

Studies have demonstrated significant differences in the composition and structure of gut microbiota among various varieties of the same species (Sun et al., 2021). Different animal species may have developed distinct gut microbiota in response to their specific environments and dietary habits. For instance, wild yaks exhibit a more intricate gut microbiota structure and higher species diversity compared to domestic yaks, reflecting their adaptation to the complex outdoor diet and habitat (Zhu et al., 2023). Similarly, Diqing Tibetan pigs display greater diversity and abundance of gut microbiota than Diannan small ear pigs (Guan et al., 2023). The challenging environment and nutrient scarcity in the Qinghai-Tibet Plateau necessitate a diverse gut microbiota in Diqing Tibetan pigs to fulfill their nutritional and energy requirements during growth. These findings indicated the significant influence of species type on gut microbiota. Shannon and Simpson indices are commonly used to evaluate microbial diversity, while Chao1 and ACE indices represent microbial species abundance (Dong et al., 2021). In this study, we found that the Chao1 and ACE indices of gut bacterial community in WH were significantly higher than those in GZH, indicating a higher bacterial abundance in WH. However, the Chao1 and ACE indices of the gut fungal community in GZH was significantly higher than that of WH. Previous research suggests that a higher diversity and abundance of gut microbiota can support more complex intestinal functions like digestion, absorption, metabolism, and immunity (Sun et al., 2022; Wang et al., 2020). GZH and WH may have evolved their own unique gut microbiota to adapt to their surroundings and diet. Moreover, we further explored the differences in intestinal structure between GZH and WH using PCoA analysis. The results showed that the structure of both gut bacterial and fungal communities was significantly different between the two breeds. These results fully demonstrate the differences in gut microbiota between the two types of horses.

Previous studies have demonstrated that *Firmicutes*, *Bacteroidota*, *Ascomycota*, and *Neocallimastigomycota* are the predominant bacterial and fungal components of the gut microbiota, and their members all play crucial roles in intestinal homeostasis and function (Huang et al., 2018; Wang et al., 2022; Zuo and Ng, 2018). It has been reported that the core bacterial and fungal species in mammals are generally stable, with changes primarily occurring in their abundance (Hubert et al., 2019; Li D. et al., 2023). The major bacterial and fungal phyla observed in WH and GZH were consistent, suggesting stability in the composition of these phyla. Furthermore, these phyla are commonly abundant in other mammals like pigs, cattle, and sheep (Kim et al., 2021). Studies have indicated that the members of *Firmicutes* possesses genes associated with biosynthesis and membrane transport (Singh and Rao, 2021). Furthermore, these members are capable of synthesizing various B vitamins, which are essential for anti-inflammatory properties and enhancing intestinal barrier function (Bellerba et al., 2021). Moreover, the *Bacteroidetes* has been found to utilize diverse dietary soluble polysaccharides and contains genes responsible for secreting vitamins and coenzymes (Hao et al., 2021). Both *Firmicutes* and *Bacteroidetes* are integral in mammalian digestion and nutrient absorption. Studies have shown that the abundance of *Firmicutes* increases in areas with harsher environments, accompanied by an increase in the proportions of *Firmicutes* and *Bacteroidetes*. In this study, GZH exhibited a higher abundance of *Firmicutes* compared to WH, while showing lower abundance of *Bacteroidetes*. This indicates that GZH have higher proportions of *Firmicutes* and *Bacteroidetes* than WH. These microbial compositions may play a role in aiding GZH in adapting to their intricate local environment and meeting their nutritional requirements.

To further investigate the changes in gut microbiota between GZH and WH, we utilized Metastats and LEfSe analyses to identify distinct bacteria and fungi. We observed that the GZH exhibited richness in *Bacillus*, *Bifidobacterium*, *Christensenellaceae_R_7_group*, *Lachnospiraceae_XPB1014_group*, *Lactococcus*, *Limosilactobacillus*, *Prevotella_7*, *Prevotellaceae_UCG_001*, *Ruminiclostridium*, while the *Lachnospiraceae_AC2044_group, Prevotellaceae_UCG_003*, *Ruminococcus*, *Weissella*, *Lachnospiraceae_UCG_009*, *Lachnospiraceae_UCG_007*, and *Oscillospira* were enriched in the WH. Studies have indicated that *Bacillus* and *Lactococcus* can synthesize broad-spectrum antibacterial compounds effective against various pathogenic bacteria (Chu et al., 2019; Horng et al., 2019). Moreover, they are also commonly used as feed additives to help maintain intestinal homeostasis, enhance function, and improve production efficiency (Mazanko et al., 2022; Zhang et al., 2021). *Christensenellaceae* have been found to produce several digestive enzymes associated with feed digestibility, underscoring their significance in growth and development (Tavella et al., 2021). *Prevotella* and *Prevotellaceae* are crucial for intestinal digestion and absorption, particularly in breaking down hemicellulose, pectin, and complex carbohydrates (Liu et al., 2018). *Bifidobacterium*, a prevalent beneficial gut bacterium, offers multiple advantages such as balancing gut microbiota, boosting immunity, and preventing diarrhea (Bo et al., 2020; Zhang et al., 2024). Moreover, it produces short-chain fatty acids and antimicrobial peptides that inhibit harmful bacteria and enhance the gut environment (Lim and Shin, 2020). *Ruminococcus* demonstrates the capacity to generate organic acids, degrade cellulose, and starch (Hong et al., 2022). Research has demonstrated that *Ruminiclostridium* lowers the occurrence of gastrointestinal issues and is linked to improved growth performance (Ravachol et al., 2015). *Lachnospiraceae*, recognized as beneficial gut bacteria, have shown an inverse relationship with intestinal inflammation (Huang et al., 2023). *Oscillospira* can metabolize host glycans to produce butyrate and short-chain fatty acids, suggesting its potential in treating inflammatory bowel disease (Gophna et al., 2017). *Weissella* has been associated with various health benefits for the host, including enhanced antioxidant capacity, disease resistance, and growth performance, as well as maintaining liver health and reducing fat accumulation (Quintanilla-Pineda et al., 2024). Both GZH and WH harbor different beneficial bacteria, which may may help them achieve their respective complex intestinal functions.

Gut microbiota play a crucial role in substance metabolism, digestion, absorption, mucosal immunity, disease prevention and control, and maintaining the intestinal barrie (Cheng et al., 2018; Li et al., 2024). Bacteria and fungi in the intestine work together to create a complex microbial system through various interactions, ultimately contributing to a range of intestinal functions and maintaining intestinal homeostasis. Thus, we further analyzed the network interactions between different bacteria or fungi and perform functional predictions for the differential bacteria. The results of network interaction analysis of gut microbiota indicate that various bacteria or fungi influence the functions of one another through complex interactions, thus amplifying the effects of different microorganisms on intestinal homeostasis and host health. The functional prediction results of gut microbiota revealed a significantly higher abundance of the digestive system in WH compared to GZH. Increased digestive system abundance may contribute to WH having a more stronger digestive system to achieve complex material digestion and energy needs. Glycans are intricate polymers composed of multiple monosaccharide molecules linked by covalent bonds. They play a crucial role in various biological processes such as development, aging, immune recognition, and cancer (Jian et al., 2020; Purushothaman et al., 2023). Taking glucan as an example, it plays a crucial role in replenishing energy, regulating gut microbiota, enhancing immunity, improving liver function, and aiding in lowering blood sugar levels (Velikonja et al., 2019). The robust glycan biosynthesis and metabolism abilities observed in WH could potentially help in storing energy and maintaining overall host health.

## Conclusion

In conclusion, this study delves into the variations in gut bacterial and fungal communities among different horse breeds. The findings reveal notable distinctions in the composition and structure of intestinal microbiota between GZH and WH. Moreover, GZH display higher abundance of gut fungal community, whereas WH showcase stronger digestive systems and glycan biosynthesis and metabolism. This research identifies distinct gut bacterial and fungal communities in both GZH and WH, potentially aiding in their adaptation to specific diets and environments.

## Data Availability

The original sequence data was submitted to the Sequence Read Archive (SRA) (NCBI, USA) with the accession no. PRJNA1116585.
